# PLGA Biodegradable Nanoparticles Containing Perphenazine or Chlorpromazine Hydrochloride: Effect of Formulation and Release

**DOI:** 10.3390/ijms151223909

**Published:** 2014-12-22

**Authors:** Mohammed Halayqa, Urszula Domańska

**Affiliations:** 1 Department of Physical Chemistry, Faculty of Chemistry, Warsaw University of Technology, Noakowskiego 3, Warsaw 00-664, Poland; E-Mail: mhalayqa@ch.pw.edu.pl; 2 Thermodynamic Research Unit, School of Engineering, University of KwaZulu-Natal, Howard College Campus, King George V Avenue, Durban 4041, South Africa

**Keywords:** perphenazine, chlorpromazine hydrochloride, psychotic drugs, experimental nanomedicine, PLGA, physicochemical properties of nanoparticles, controlled drug delivery

## Abstract

In our study, poly(dl-lactide-co-glycolide) (PLGA) nanoparticles loaded with perphenazine (PPH) and chlorpromazine hydrochloride (CPZ-HCl) were formulated by emulsion solvent evaporation technique. The effect of various processing variables, including PLGA concentration, theoretical drug loading, poly(vinyl alcohol) (PVA) concentration and the power of sonication were assessed systematically to obtain higher encapsulation efficiency and to minimize the nanoparticles size. By the optimization formulation process, the nanoparticles were obtained in submicron size from 325.5 ± 32.4 to 374.3 ± 10.1 nm for nanoparticles loaded with PPH and CPZ-HCl, respectively. Nanoparticles observed by scanning electron microscopy (SEM) presented smooth surface and spherical shape. The encapsulation efficiency of nanoparticles loaded with PPH and CPZ-HCl were 83.9% and 71.0%, respectively. The drug loading were 51.1% and 39.4% for PPH and CPZ-HCl, respectively. Lyophilized nanoparticles with different PLGA concentration 0.8%, 1.3% and 1.6% (*w*/*v*) in formulation process were evaluated for *in vitro* release in phosphate buffered saline (pH = 7.4) by using dialysis bags. The release profile for both drugs have shown that the rate of PPH and CPZ-HCl release were dependent on a size and amount of drugs in the nanoparticles.

## 1. Introduction

In recent years, there has been an increased interest in formulation nanoparticles (NPs) loaded selected drugs for use in drug delivery. Particularly, polymeric NPs have obtained increasing attention in pharmaceutics and in the fields of drug delivery. The polymeric NPs show high effectiveness as drug delivery agents due to their specific properties, such as extending the drug release, decreasing drug degradation, increasing bioavailability and reducing drug toxicity [1,2,3,4].

Among the polymeric NPs, the most widely used is poly(dl-lactide-co-glycolide) (PLGA). PLGA is one of the most successfully investigated drug delivery system because of its tissue compatibility, biodegradability into metabolite monomers, glycolic acid and lactic acid, low toxicity as well as because these two monomers are endogenous and simply metabolized by the body via the Krebs cycle [1,3,4,5,6,7,8]. The new high throughput screening method has just been developednow by us to measure release profiles of piroxicam from polylactic acid or PLGA NPs [8]. This method could present a large number of advantages for formulation screening purpose in the early stage of drug research. Several specific drugs were tested as PLGA NPs with drug loading 10% to 30% (*w*/*w*) [9]. For low molecular mass (*M*_W_) (58,000) of PLGA, typical particle sizes ranged from 60–200 nm were obtained with release times of about seven weeks (which also depended on the kind of drug) [9]. The PLGA polymer was also used for the nanoformulation of levofloxacin, for which the average size of NPs obtained for different conditions was 268 nm [10]. Using different pH and optimization methods of NPs, fabrications with theoretical drug loading ranged from 5% to 20% (*w*/*w*), the NPs of 5-fluorouracil in PLGA (*M*_W_ 15,000) sized in range 189.2 and 233.6 nm were obtained [11]. The process of praziquantel NPs formation using PLGA (*M*_W_ 40,000–100,000), with drug loading 10% to 30% (*w*/*w*) and emulsion formation, the particle sizes were obtained in a range of 250 to 340 nm [12].

Phenothiazine derivatives have been used since the 1950s as depressants and antipsychotic drugs. They have also been announced to have anti-cancer and anti-infective activities [13]. Phenothiazine drugs, such as chlorpromazine and fluphenazine, together with haloperidol are the only antipsychotics listed as “essential drugs” by the World Health Organization (WHO) (2011) [14]. The systematic review and meta-analysis of the older literature on first generation antipsychotics is presented as well [14].

The perphenazine (PPH) and chlorpromazine hydrochloride (CPZ-HCl) have low oral bioavailability of 30%–50% because of their comprehensive first pass metabolism in liver. These drugs can be useful as a long-term medication forpsychiatric disorders. Nasal drug delivery could be used to improve therapeutic efficacy through avoidance of hepatic first pass metabolism, which is large in the case of these drugs [15]. TheCPZ-HCl NPs with PLGA grafted with chitosan on the surface were formulated under different variables giving average particle sizes ranged from 306.1 to 700.0 nm [15]. Chitosan, used in this experiment, enhanced mucoadhesion property of NPs. Another aspect that should be considered in psychotic drugs treatment, is the targeting of drugs to the brain, as the blood–brain barrier (BBB) prevents most substances from freely penetrating and diffusing into the central nervous system from the bloodstream in order to keep brain homeostasis. Various methods have been used for providing drugs to the central nervous system, among them intranasal administration, which involves avoiding the blood–brain barrier and delivering therapeutic drugs directly to the central nervous system. Therefore encapsulation drugs, such as PPH and CPZ-HCl loaded-PLGA NPs, as a nanocarier could enhance the therapeutic efficacy and would also provide brain targeting and sustained release of drug within the brain. PPH is derivative of phenothiazine and is known to be used to treat psychosis. The drug affects the central nervous system. It belongs to the first generation neuroleptics. It shows a strong antipsychotic activity. This drug has a stronger influence on behavior than other phenothiazines due to having in their structure piperazine group [16]. Clinical indications for PPH include: Schizophrenia, psychotic depression, hallucinations and hyperemesis gravidarum. An interesting method using phenothiazines, including chlorpromazine delivery system with the isolated plasma derived chylomicron system, was presented by Shahnaz *et al*. [17]. With this method, it was possible to obtain particle sizes lower than 200 nm [17]. The mechanism of the intestinal lymphatic transfer of lipophilic drugs with chylomicrons was described as well [18].

The solubility of CPZ-HCl in three important solvents for drugs, water, ethanol and 1-octanol using the dynamic method and UV-VIS method at constant pH, was measured by us in our earlier work [19]. Also new association constants and corresponding p*K*a values of CPZ-HCl was obtained with Bates-Schwarzenbach method at temperature 298.15 K in the buffer solutions [19].

In the present study, the formulation of PLGA nanoparticles loaded with PPH and CPZ-HCl was provided and the impact of formulation variables on size distribution, polydispersity, encapsulation efficiency and the drug loading of NPs were investigated. The second objective of this work was to examine the profile release of nanoparticles loaded with PPH and CPZ-HCl. The novelty of this work is to analyze the formulation parameters of the known emulsion solvent evaporation method and PLGA/PVA polymer system to phenothiazine derivatives and minimize the size of the NPs.

## 2. Results and Discussion

### 2.1. Preparation and Characterization of Poly(dl-lactide-co-glycolide) (PLGA) Nanoparticles (NPs)

In our study we have investigated PPH and CPZ-HCl loaded nanoparticles as a drug carrier. Both drugs were formulated by emulsion-solvent evaporation method. PPH was prepared in aqueous phase, which was buffered to pH = 7, whereas the aqueous phase of CPZ-HCl was buffered to pH = 9 in order to prevent the CPZ-HCl from diffusion to the aqueous phase. In our study, the effect of variable parameters and conditions were investigated for the preparations of NPs: the theoretical drug loading (TDL) and poly(vinyl alcohol) (PVA) concentration in the formulation, the PLGA content and the power of sonication. Only one, or two parameters were changed in each series of experiments. The investigated compounds are listed in Table 1.

**Table 1:** Investigated compounds: Name, abbreviation, structure, and molar mass.

Name of Compound	Structural Formula	*M*_W_ (g∙mol^−1^)
Perphenazine (PPH)	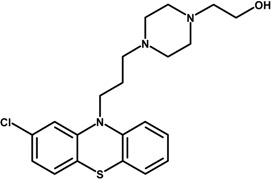	403.97
Chlorpromazine hydrochloride (CPZ-HCl)	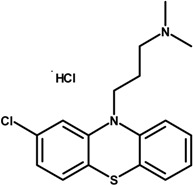	355.30
Poly(dl-lactide-co-glycolide) (PLGA: 50:50)	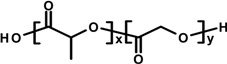	54,000 69,000

### 2.2. Size Distribution and Polydispersity

In this study, the effect of four preparation variables on diameter and polydispersity (PDI) of NPs was studied. The experimental conditions and the obtained results are shown in Table 2. It is easy to observe that an increase of PLGA concentration increases the diameter of NPs for both drugs. The size of nanoparticles containing PPH increases from 340.5 ± 17.8 to 382.5 ± 36.5 nm with an increase of amount of PLGA concentration from 0.8% to 1.3% (*w*/*v*). The same effect was observed for the CPZ-HCl. An increase of PLGA concentration from 0.8% to 1.6% (*w*/*v*) increases the size of NPs from 374.3 ± 10.1 to 426.0 ± 19.4 nm, respectively. This effect was observed in many earlier studies [12,20,21]. It could be explained by an increase of the amount of PLGA in organic phase, which makes the solution more viscous and as a result reduces the net shear stress and makes bigger droplets. In addition, an increasing of the viscosity of the organic phase decreases the dispersion of organic phase into the aqueous phase, resulting in the larger size of NPs. The polydispersity of the NPs is small and indicates the narrow size distribution of the NPs.

The results in Table 2 shows the effect of the TDL on the size of NPs, different TDL selected with maintaining constant amount of PLGA in organic phase. The mass of drug in relation to the mass of PLGA was varied between 10% and 30% (*w*/*w*).

**Table 2:** Effect of poly(dl-lactide-co-glycolide) (PLGA), the theoretical drug loading (TDL), poly(vinyl alcohol) (PVA) and power of sonication (Power Son.) on the on the nanoparticle’s (NPs) size and polydispersity (PDI). (Numbers in bold are discussed below).

PLGA (%)	TDL (%)	PVA (%)	Power Son. (%)	PPH		CPZ-HCl	
				NPs (nm)	PDI	NPs Size (nm)	PDI
**0.8**	20	0.5	30	340.5 ± 17.8	0.112 ± 0.016	374.3 ± 10.1	0.149 ± 0.003
**1.3**	**20**	**0.5**	**30**	**382.5 ± 36.5**	**0.119 ± 0.022**	**374.3 ± 10.1**	**0.149 ± 0.003**
**1.6**	20	0.5	30	376.6 ± 28.6	0.107 ± 0.034	426.0 ± 19.4	0.123 ± 0.037
1.3	**10**	0.5	30	367.2 ± 22.7	0.138 ± 0.013	382.4 ± 10.2	0.178 ± 0.030
1.3	**30**	0.5	30	397.1 ± 31.1	0.149 ± 0.048	397.9 ± 28.8	0.098 ± 0.016
1.3	20	**0.3**	30	–	–	476.7 ± 11.2	0.243 ± 0.032
1.3	20	**0.7**	30	325.5 ± 32.4	0.105 ± 0.020	395.2 ± 24.4	0.150 ± 0.016
1.3	20	0.5	**50**	363.0 ± 30.9	0.109 ± 0.010	403.5 ± 12.2	0.148 ± 0.013
1.3	20	0.5	**70**	419.1 ± 23.4	0.158 ± 0.004	443.0 ± 11.1	0.183 ± 0.030

An increase in the amount of the drug in the organic phase increases the size of the NPs. The NPs loaded with PPH show the regular increase of size with an increase of the drug contents in the organic phase, whereas the size of NPs loaded with CPZ-HCl changes irregularly: First decreases and then increases with an increase of the drug concentration. It can be observed that an increase of the density of organic phase results in the decrease of the dispersion of the organic phase into aqueous phase, which results in an increase of the NPs size. This effect is very similar to that observed in the PLGA increasing concentration. An increase of the size of NPs for both drugs was more obvious in the case of an increase of the amount of PLGA in organic phase than those of the increase of the drug contents.

The effect of the concentration of PVA in the solution and as a result in the aqueous phase on the size and PDI of NPs was studied. It can be observed that for PPH the high concentration of a surfactant leads to reduction of the mean size of NPs (from 382.5 ± 36.5 to 325.5 ± 32.4 nm), when the concentration of PVA increases from 0.5% to 0.7% (*w*/*v*). The same consequence was observed for NPs loaded with CPZ-HCl, when the concentration of PVA = 0.3% (*w*/*v*) the NPs obtained had a mean size 476.7 ± 11.2 nm. It was also observed that the NPs loaded with CPZ-HCl have a wide range of PDI. When the PVA concentration increases from 0.3% to 0.7% (*w*/*v*), the PDI decreases from 0.243 ± 0.032 to 0.150 ± 0.016, respectively. At high concentration of PVA, the surfactant play remarkable role in the reducing of the interfacial tension of the organic/aqueous interface [22], which results in an increasing of the net share stress and smaller droplets formed during the emulsification process. It also results in the decreasing of the size of nanoparticles with an increase of PVA concentration [23,24,25]. In addition, the higher concentration of PVA in the aqueous phase would protect the forming droplets in the emulsion process and can avoid the globules from coalescence [20,21,26]. On the other hand, an increase in the PVA concentration leads to an increase in the density of the aqueous phase and decreases the net share stress in the emulsification process, which results in an increase of the mean diameter of the NPs [27]. Our results indicated that the stabilizing effect of PVA was dominated and resulted in the smallest size of NPs and were accomplished with an increase of PVA concentration.

The power of sonication also has a remarkable effect on the droplets size. With an increase of power sonication, the nanoparticles sizes are appreciably larger and the PDI increases (see Table 2). It can be explain by the possible raise of temperature of solution as a result of the increase of the power of sonication. As a consequence, the coalescence of droplets and an increase of the NPs size may be observed.

### 2.3. Drug Efficiency and Drug Loading

The procedure for measuring encapsulation efficiency and drug loading are the same for both drugs. The indirect method was carried out by measuring the amount of drug uncapsulated in NPs. Freshly prepared NPs solution was centrifuged at 6000 rpm/min for 20 min to sediments of solid NPs. Next, the solution of drug in the supernatant was analyzed, assuming that drug not present in the supernatant was capsulated into PLGA NPs [28,29]. The encapsulation efficiency (EE) and the drug loading (DL) were calculated from the Equations (1) and (2).

(1) $$\text{EE(\%)}=\frac{\text{Amount of drug encapsulation}}{\text{amount of drug}}\times 100$$

(2) $$\text{DL(\%)}=\frac{\text{Amount of drug (mg) in NPs}}{\text{100 mg of NPs}}\times 100$$

The results for the effect of PLGA, theoretical drug loading, PVA and the power of sonication are listed on Table 3. An increase of the PLGA concentration leads to an increase of the drug encapsulation and drug loading for both investigated drugs. These results are expected due to an increase of viscosity of organic phase. An increase in density can avoid the drug diffusion from organic phase to the aqueous phase. Additionally, increasing of viscosity of the solution results in an increase of the NPs size. This may be related to an increase in amount of drugs in NPs [29]. On the other hand, the aqueous phase containing CPZ-HCl was buffered to pH = 9, which kept the drug from being lost. In this pH the CPZ-HCl was insoluble in water.

**Table 3:** Effect of poly(dl-lactide-co-glycolide) (PLGA), the theoretical drug loading (TDL), poly(vinyl alcohol) (PVA) and power of sonication (Power Son.) on encapsulation efficiency (EE) and drug loading (DL). (Numbers in bolt are discussed below).

PLGA (%)	TDL (%)	PVA (%)	Power Son. (%)	PPH		CPZ-HCl	
				EE (%)	DL (%)	EE (%)	DL (%)
**0.8**	20	0.5	30	46.6	10.2	44.8	16.1
**1.3**	20	0.5	30	**66.3**	**23.8**	**55.2**	**20.1**
**1.6**	20	0.5	30	71.6	25.5	61.6	22.1
1.3	**10**	0.5	30	39.9	6.3	56.9	9.5
1.3	**30**	0.5	30	83.9	51.1	64.4	39.4
1.3	20	**0.3**	30	-	-	71.0	25.7
1.3	20	**0.7**	30	56,4	20,8	46.7	16.9
1.3	20	0.5	**50**	56.4	20.8	66.6	24.1
1.3	20	0.5	**70**	65.2	23.3	65.1	23.2

An increase in the amount of drug in the organic phase has remarkable influence on the EE and DL [25]. The EE increases from 39.9% to 83.9% (*w*/*w*) with an increase of TDL from 10% to 30% (*w*/*w*) for NPs containing PPH. For the nanoparticles loaded with CPZ-HCl, there was only a small increase in EE with an increase of TDL. An increase of concentration of drug in the organic phase causes an increase of the viscosity of solution. As a consequence, the larger NPs are formulated.

In contrast, an increase of the PVA concentration decreases the entrapment efficiency, which results in the decreasing of NPs size.

As shown in Table 3, the power of sonication had no significant effect on the DL and EE for both drugs. These results are in the accordance with published earlier [25].

### 2.4. Morphology of Nanoparticles

**Figure 1:**
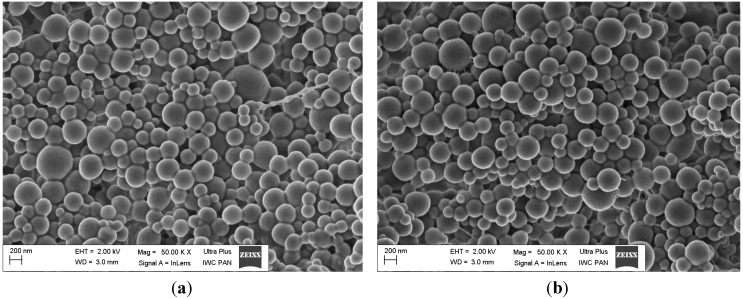

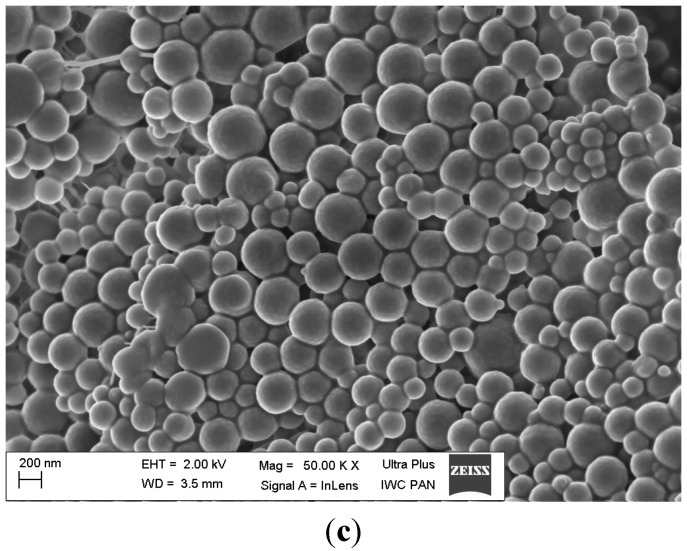
SEM images of PPH-loaded PLGA prepared with PLGA concentration (**a**) 0.8% (*w*/*v*); (**b**) 1.3% (*w*/*v*); and (**c**) 1.6% (*w*/*v*) at TDL 20% (*w*/*w*).

Morphology of the NPs was investigated by scanning electron microscopy (SEM). Figure 1a–c show the images for PPH-loaded PLGA nanoparticles with different concentration of PLGA of 0.8%, 1.3% and 1.6% (*w*/*v*), respectively. The NPs show spherical shape and smooth surface and absence of different NPs size. In our study we measured the mean size diameter by Dynamic Light Scattering (DLS) technique, which based on measuring the *Z*-Average of nanoparticles. There are submicron particles with diameter size 205.2 nm and bigger particles with diameter size 391.8 nm (for PLGA concentration 1.3% (*w*/*v*)).

**Figure 2:**
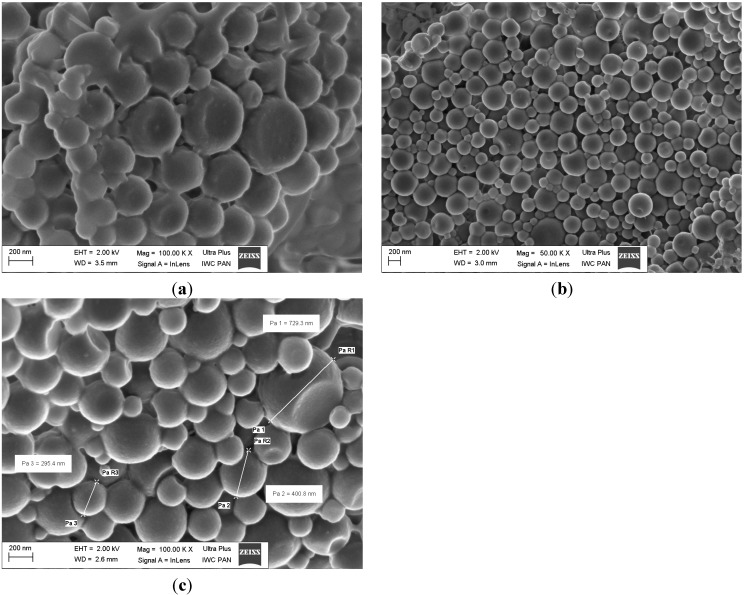
SEM images of CPZ-HCL-loaded PLGA prepared with PLGA concentration (**a**) 0.8% (*w*/*v*); (**b**) 1.3% (*w*/*v*); (**c**) 1.6% (*w*/*v*) at TDL 20% (*w*/*w*).

Figure 2a–c show SEM images for CPZ-HCl nanoparticles with variables of PLGA concentration of 0.8%, 1.3% and 1.6% (*w*/*v*), respectively. The NPs prepared with PLGA concentration of 1.3% and 1.6% (*w*/*v*), respectively, present spherical shape and smooth surface. In contrast, the NPs prepared with PLGA concentration 0.8% (*w*/*v*) have rough exterior and are spherical shape but the globules are coalesced to each other. The coalescence of particles is probably caused by the uncapsulated drug, or surfactant that had not been fully removed in final stage of the preparation.

### 2.5. In Vitro Drug Release

**Figure 3:**
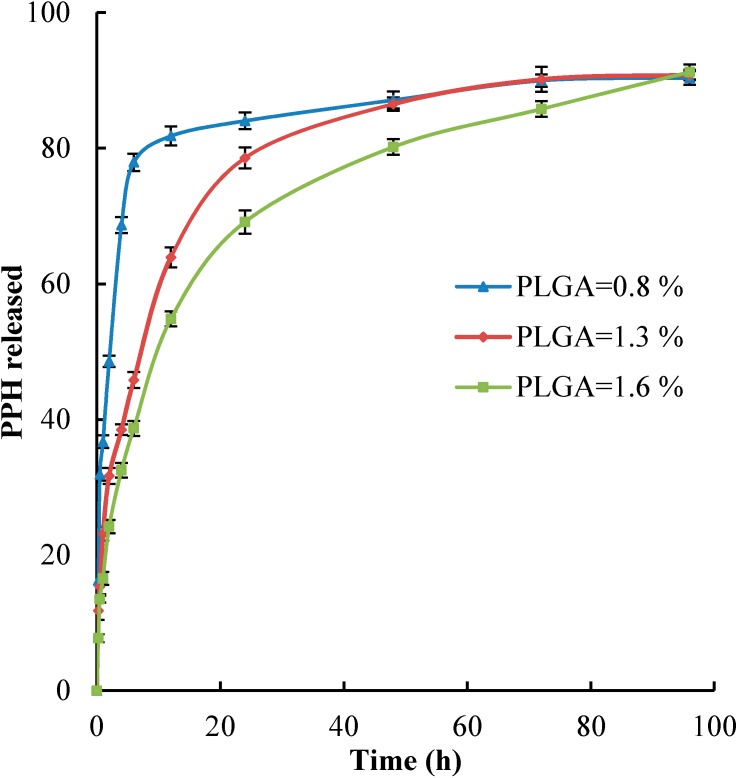
The *in vitro* PPH release profile from NPs at *T* = 310.15 K in phosphate buffered saline, pH 7.4 for the same amount of drug: Results for different concentration of PLGA (*w*/*v*): (▲) 0.8%, (♦) 1.3%, (■) 1.6%.

The *in vitro* drug release from nanoparticles was carried out in phosphate buffered saline in pH 7.4 at *T* = 310.15 K. The *in vitro* release of PPH from NPs for different PLGA concentrations 0.8%, 1.3% and 1.6% (*w*/*v*) is presented in Figure 3. The drug release profile shows biphasic character containing a rapid initial burst release followed with a sustained release of PPH with PLGA concentration of 1.3% and 1.6% (*w*/*v*). The burst release was found for the first 24 h. The high release at the burst time related to the releasing drug, which was absorbed or entrapped on the surface of NPs. The second phase of PPH release was related to the diffusion mechanism. The profile release of PPH containing PLGA concentration 0.8% (*w*/*v*) was different and show only burst release.

**Figure 4:**
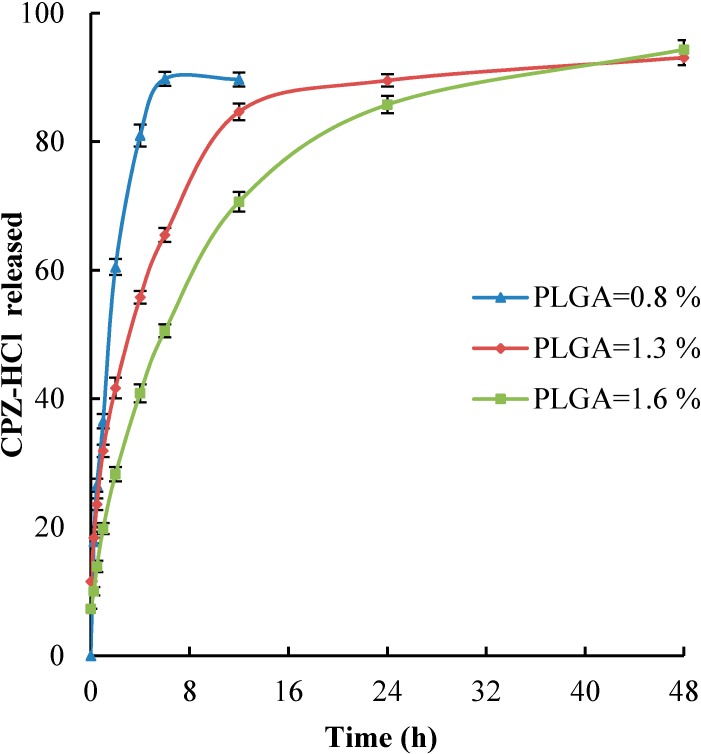
The *in vitro* CPZ-HCl release profile from NPs at *T* = 310.15 K in phosphate buffered saline, pH 7.4 for the same amount of drug: Results for different concentration of PLGA (*w*/*v*): (▲) 0.8%, (♦) 1.3%, (■) 1.6%.

The profile release of CPZ-HCl-loaded nanoparticles with variable PLGA concentration 0.8%, 1.3% and 1.6% (*w*/*v*) is shown in Figure 4. The release profile of CPZ-HCl-loaded NPs with PLGA concentration of 1.6% and 1.3% (*w*/*v*) exhibited similar release and characterization by an initial burst in which CPZ-HCl was highly released during 16 h and then followed by a slow release. The CPZ-HCl release from NPs with PLGA concentration 0.8% (*w*/*v*) was very rapidly released and almost 90% of drug was released during six hours. In the case of NPs loaded with CPZ-HCl, the drug was faster released than that for NPs loaded with PPH. It is probably related to the pH of aqueous phase in formulation process. At high pH the polymer undergoes degradation during processing resulting in a reduction in molecular weight of the PLGA [30]. The profile release of NPs loaded with CPZ-Cl was similar to the profile release of NPs loaded with PPH, which was formulated under the same condition as NPs loaded with CPZ-HCl [31].

An important phenomenon recognized is the slower release for the both drugs taking place for the NPs that have bigger particle size and higher encapsulation efficiency. The NPs size has influence effect on dissolution rate of NPs, which increases with the decreasing of the NPs size due to an increase of available surface area [32]. The effect of encapsulation efficiency on drug release profile was similar to that reported earlier in the open literature [33].

## 3. Experimental Section

### 3.1. Chemicals and Reagents

Perphenazine (PPH) (CAS number 58-39-9) purchased from Sigma-Aldrich, Xian, China and chlorpromazine hydrochloride (CPZ-HCl) (CAS number 69-09-0) were purchased from Sigma-Aldrich, Eichenzel, Germany. PLGA as a copolymer ratio of dl-lactide to glycolide of 50:50 (*M*_W_ 54,000–69,000 g∙mol^−1^) was purchased from Sigma-Aldrich, Steinhein, Germany. The poly(vinyl alcohol) (PVA) (95% hydrolysis degree and *M*_W_ 95,000 g∙mol^−1^), supplied from Across-Organics, Morris Plains, NJ, USA (CAS number 9002-89-5) was used as the surfactant in the emulsification process. Visking dialysis bags with pore size of 12 kDa were obtained from Sigma-Aldrich, Germany. Chemicals used: Dichloromethane (CAS number 75-09-2, 0.999 mass fraction) and acetonitrile (CAS number 75-05-8, 0.99 mass fraction) for preparing buffer solution pH = 9, which was used in aqueous phase for CPZ-HCl preparation and pH = 7.4, which used as medium in drug release. The dipotassium hydrogen phosphate (CAS number 7758-11-4, 0.999 mass fraction) purchased from Sigma-Aldrich, Steinhein, Germany, potassium hydroxide (CAS number 1310-58-3, 0.995 mass fraction purity), and potassium dihydrogen phosphate (CAS number 7758-11-4, 0.995 mass fraction purity), all purchased from POCH, Gliwice, Poland were used in experiment.

Solvents were analytical grade and were used without further purification. Twice distilled and degassed water, deionized and filtered with Milipore Elix 3, was used for the aqueous solutions of drugs.

### 3.2. Preparation of Nanoparticles

The Nanoparticles loaded with PPH or CPZ-HCl were formulated by an emulsion-solvent evaporation method and by using PVA as a surfactant. The preparation conditions were the same for both drugs; shortly 10 mg of drug and 40 mg of PLGA were dissolved in 3 mL of methylene chloride to achieve TDL 20% (*w*/*w*) of drug with PLGA in organic phase. The organic phase (3 mL) was then added dropwise into 10 mL 0.5% (*w*/*v*) aqueous solution of PVA over 2 min under constant stirring. In the case of CPZ-HCl, the aqueous phase was buffered to pH = 9.0 with the phosphate buffer, in order to minimize drug loss. The resulting mixture was then sonicated with the help of probe sonicator set at level 30% of power 70 W for 3 min (HD 2070, Bandelin Sonopuls, Germany). The amount of methylene chloride was evaporated during 15 min using a rotative evaporator under partial vacuum. The NPs were isolated by centrifugation (6000 rpm, 20 min, Hitachi, Tokyo, Japan) and washed twice with double-distilled water. The NPs were freshly used or lyophilized for the further investigation.

### 3.3. Size Distribution and Polydispersity Index

The NPs size and polydispersity index were measured using dynamic light scattering technique (DLS) on the zetasizer Nano (Malvern Instruments, Malvern, UK). The freshly prepared and purified suspension of NPs was diluted in the distilled water and the mixture was sonicated during 2 min to separate the bigger aggregates. The analysis was carried out at a scattering angle of 90° and at a temperature of *T* = 298.15 K. The *Z*-average diameter and polydispersity index were calculated using Malvern software. For statistical analysis all samples were measured in triplicate and the average values and standard deviation of the measurements were calculated.

### 3.4. Scanning Electron Microscopy

The morphology of NPs was analyzed using scanning electron microscopy (SEM, Ultra Plus, Zeiss, Oberkochen, Germany) at operating voltage of 2 kV. The samples of lyophilized NPs were initially seated on metal stubs and cut by razor blade and then dried under vacuum. Afterwards, samples where coated with carbon layer using a sputter coater (BAL-TEC SCD 005 Sputter Coater, Capovani Brothers Inc., Scotia, NY, USA).

### 3.5. Assay of Encapsulation

The amount of drug encapsulated in NPs was determined spectrophotometrically using the Spectrometer UV-vis Lambda 25 (Perkin Elmer Life and Analytical Sciences, CT, Shelton, USA) at chosen wavelength λ = 255 nm, λ= 257 nm for PPH or CPZ-HCl, sequentially. The reference cuvette was filled with the mixture of acetonitrile and water (1:1 *v*/*v*). The linear calibration curve for PPH and CPZ-HCl was obtained in the same mixture of acetonitrile with water (1:1 *v*/*v*) in the range from 3 to 15 μg/mL. The overall experimental uncertainty for the temperature was estimated to be ±0.05 K. Photometric accuracy (NIST 930D Filter 1A) obtainable with UV-Vis Spectrophotometer is ±0.001 A and repeatability ≤0.001 A. The uncertainty in composition was 1 × 10^−6^ mol∙dm^−3^. Many series of UV spectra were recorded at *T* = 298.15 K, and for each sample the obtained peaks were analyzed. The calibration curves for the chosen wavelength λ = 255 nm and λ = 257 nm were used for PPH and CPZ-HCl, respectively.

### 3.6. In Vitro Drug Release

The *in vitro* release study was performed by dialysis bag diffusion technique. Lyophilized NPs were evaluated for *in vitro* release in phosphate–buffered saline (pH = 7.4) by using dialysis bags. First, NPs were resuspended and dispersed by sonication in 1 mL of buffer and then poured in dialysis bags. The dialysis bags were immersed into 80 mL of release medium (phosphate buffered saline pH = 7.4) at temperature *T* = 310.15 K under magnetic stirring at 100 rpm. At predetermined time intervals, aliquots of 1 mL were withdrawn and analyzed spectrophotometrically as described in section 3.5 to determined the amount of drug released.

## 4. Conclusions

In this paper, two phenothiazine-derivative drugs, namely perphenazine (PPH) and chlorpromazine hydrochloride (CPZ-HCl), were successfully encapsulated in PLGA as nanoparticles by emulsion solvent evaporation technique. The effect of different processing variables on nanoparticles size, polydispersity, drug loading, and encapsulation efficiency were investigated to obtain optimal nanoparticles formulation. This study has shown that the PPH and CPZ-HCl could be encapsulated into PLGA NPs, with comparatively high encapsulation efficiency of 83.9% for PPH and 71.0% for CPZ-HCl, respectively. It was proved that the small size (around 380 nm) may be obtained. The drug release profile for both drugs was similar and exhibited biphasic characters involving a rapid initial burst release followed with a sustained release for the nanoparticles with PLGA concentration 1.3% and 1.6% (*w*/*v*), while the nanoparticles with PLGA concentration 0.8% (*w*/*v*) show only burst release. Release of drugs was dependent on the particles size and on encapsulation efficiency. The release of PPH and CPZ-HCl from nanoparticles was slower from the NPs with larger size and higher encapsulation efficiency.

## References

[1] Cooper D.L., Harirforoosh S. (2014). Design and optimization of PLGA-based diclofenac loaded nanoparticles. PLoS One.

[2] Zarrabi A., Vossoughi M., Alemzadeh I., Chitsazi M.R. (2012). Monodispersed polymeric nanoparticles fabrication by electrospray atomization. Int. J. Polym. Mater..

[3] Zhou Y.Y., Du Y.Z., Wang L., Zhou J.P., Hu F.Q. (2010). Preparation and pharmacodynamics of stearic acid and poly(lactic-co-glycolic acid) grafted chitosan oligosaccharide micelles for 10-hydroxycamptothecin. Int. J. Pharm..

[4] Rinaldi S., Fortunati E., Taddei M., Kenny J.M., Armentano I., Latterini L. (2013). Integrated PLGA-Agnanocomposite systems to control the degradation rate and antibacterial properties. J. Appl. Polym. Sci..

[5] Pascolo L., Bortot B., Benseny-Cases N., Gianoncelli A., Tosi G., Ruozi B., Rizzardi K., de Martino E., Vandelli M., Severini G.M. (2014). Detection of PLG A-based nanoparticles at a single-cell level by synchrotron radiation FTIR spectromicroscopy and correlation with X-ray fluorescence microscopy. Int. J. Nanomed..

[6] Kumari A., Yadav S.K., Yadav S.C. (2010). Biodegradable polymeric nanoparticles based drug delivery systems. Colloids Surf. B.

[7] Danhier F., Lecouturier N., Vromana B., Jerome C., Marchand-Brynaert J., Ferond O., Preat V. (2009). Paclitaxelloaded PEGylated PLGA-based nanoparticles: *In vitro* and *in vivo* evaluation. J. Control. Release.

[8] Pelczarska A., Delie F., Domańska U., Carrupt P., Martel S. (2013). New high throughput screening method for drug release measurements. Eur. J. Pharm. Biopharm..

[9] Song C.X., Labhasetwar V., Murphy H., Qu X., Humphrey W.R., Shebuski R.J., Levy R.J. (1997). Formulation and characterization of biodegradable nanoparticles for intravascular local drug delivery. J. Control. Release.

[10] Kumar G., Sharma S., Shafiq N., Khuller G.K., Malhotra S. (2012). Optimization, *in vitro*-*in vivo* evaluation, and short-term torelability of novel levofloxacin-loaded PLGA nanoparticle formulation. J. Pharm. Sci..

[11] Wang Y., Li P., Peng Z., She F.H., Kong L.X. (2013). Microencapsulation of nanoparticles with enhanced drug loading for pH-sensitive oral drug delivery for the treatment of colon cancer. J. Appl. Polym. Sci..

[12] Mainardes R.M., Evangelista R.C. (2005). PLGA nanopartcles containing praziquantel: Effect of formulation variable on size distribution. Int. J. Pharm..

[13] PIENO. http://www.parkinsons-information-exchange-network-online.com/drugdb/029..

[14] Samara M.T., Cao H., Helfer B., Davis J.M., Leucht S. (2014). Chlorpromazine *versus* very other antipsychotic for schizophrenia: A systematic review and meta-analysis challenging the dogma of equal efficacy of antipsychotic drugs. Eur. Neuropsychopharmacol..

[15] Chalikwar S.S., Mene S.B., Pardeshi C.V., Belgamwar V.S., Surana S.J. (2013). Self-assembled, chitosan grafted PLGA nanoparticles for intranasal delivery: Design, development and *ex vivo* characterization. Polym.-Plast. Technol. Eng..

[16] Meltzer H.Y., Massey B.W. (2011). The role of serotonin receptors in the action of atypical antipsychotic drugs. Curr. Opin. Pharmacol..

[17] Shahnaz G., Harlt M., Barthelmes J., Leithner K., Sarti F., Hintzen F., Rahmat D., Salvenmoser W., Bernkop-Schnürch A. (2011). Uptake of phenothiazines by the harvested chylomicrons *ex vivo* model: Influence of sel-nanoemulsifying formulation design. Eur. J. Pharm. Biopharm..

[18] Aji Alexa M.R., Chackoa A.J., Josea S., Soutob E.B. (2011). Lopinavir loaded solid lipid nanoparticles (SLN) for intestinal lymphatic targeting. Eur. J. Pharm. Sci..

[19] Domańska U., Pelczarska A., Pobudkowska A. (2011). Solubility and p*K*a determination of six structurally related phenothiazines. Int. J. Pharm..

[20] Quintanar-Guerrero D., Fessi H., Allémann E., Doelker E. (1996). Influence of stabilizing agents and preparatives variables on the formation of poly(dl-lactic acid) nanoparticles by an emulsification-diffusion technique. Int. J. Pharm..

[21] Murakami H., Kobayashi M., Takeuchi H., Kawashima Y. (1999). Preparation of poly(dl-lactide-co-glycolide) nanoparticles by modified spontaneous emulsification solvent diffusion method. Int. J. Pharm..

[22] Galindo-Rodriguez S., Alleman E., Fessi H., Doelker E. (2004). Physicochemical parameters associated with nanoparticle formulation in the salting-out emulsification-diffusion and nanoprecipitation methods. Pharm. Res..

[23] Nandi A., Khakhar D.V., Mehra A. (2001). Coalescence in surfactant-stabilized emulsions subjected to shear flow. Langmuir.

[24] Tesch S., Schubert H. (2002). Influence of increasing viscosity of the aqueous phase on the short-term stability of protein stabilization emulsion. J. Food Eng..

[25] Song X., Zhao Y., Wu W., Bi Y., Cai Z., Chen Q., Li Y., Hou S. (2008). PLGA nanoparticles simultaneously loaded with vincristine sulfate and verapamil hydrochloride: Systematic study of particle size and drug entrapment efficiency. Int. J. Pharm..

[26] Kwon H.-Y., Lee J.-Y., Choi S.-W., Jang Y., Kim J.-H. (2001). Preparation of PLGA nanoparticles containing estrogen by emulsification-diffusion method. Colloids Surf. A.

[27] Budhian A., Siegel S.J., Winey K.I. (2007). Haloperidol-loaded PLGA nanoparticles: Systematic study of particle size and drug content. Int. J. Pharm..

[28] Das R.K., Kasoju N., Bora U. (2010). Encapsulation of curcumin in alginate-chitosan-pluronic composite nanoparticles for delivery to cancer cells. Nanomedicine.

[29] Fritze A., Hens F., Kimpfler A., Schubert R., Peschka-Suss R. (2006). Remote loading of doxorubicin into liposomes driven by a transmembrane phosphate gradient. Biochim. Biophys. Acta.

[30] Dunne M.M., Ramtoola Z., Corrigan O.I. (2009). Fluphanazine release from biodegradable microparticles: Characterization and modelling of release. J. Microencapsul..

[31] Domańska U., Halayqa M. (2014). Promazine Hydrochloride/PLGA biodegradable nanoparticles formulation and release. J. Phys. Chem. Biophys..

[32] Zili Z., Sfar S., Fessi H. (2005). Preparation and characterization of poly-ε-caprolactone nanoparticles containing griseofulvin. Int. J. Pharm..

[33] Huang C., Chen C., Lee Y. (2007). Synthesis of high loading and encapsulation efficient paclitaxel-loaded poly(*n*-butyl cyanoacrylate) nanoparticles via miniemulsion. Int. J. Pharm..

